# C and N metabolism in barley leaves and peduncles modulates responsiveness to changing CO_2_

**DOI:** 10.1093/jxb/ery380

**Published:** 2018-11-24

**Authors:** Fernando Torralbo, Rubén Vicente, Rosa Morcuende, Carmen González-Murua, Iker Aranjuelo

**Affiliations:** 1 Department of Plant Biology and Ecology, University of the Basque Country (UPV/EHU), Bilbao, Spain; 2 Instituto de Agrobiotecnología (IdAB)-CSIC, Avenida de Pamplona, Mutilva Baja, Spain; 3 Abiotic Stress Department, Institute of Natural Resources and Agrobiology of Salamanca, IRNASA-CSIC, Salamanca, Spain; 4 Max Planck Institute of Molecular Plant Physiology, Am Mühlenberg, Potsdam, Germany

**Keywords:** Barley, carbohydrates, elevated CO_2_, gene expression, N assimilation, peduncle, photosynthetic down-regulation, sink–source

## Abstract

Balancing of leaf carbohydrates is a key process for maximising crop performance in elevated CO_2_ environments. With the aim of testing the role of the carbon sink–source relationship under different CO_2_ conditions, we performed two experiments with two barley genotypes (Harrington and RCSL-89) exposed to changing CO_2_. In Experiment 1, the genotypes were exposed to 400 and 700 ppm CO_2_. Elevated CO_2_ induced photosynthetic acclimation in Harrington that was linked with the depletion of Rubisco protein. In contrast, a higher peduncle carbohydrate-storage capacity in RSCL-89 was associated with a better balance of leaf carbohydrates that could help to maximize the photosynthetic capacity under elevated CO_2_. In Experiment 2, plants that were grown at 400 ppm or 700 ppm CO_2_ for 5 weeks were switched to 700 ppm or 400 ppm CO_2_, respectively. Raising CO_2_ to 700 ppm increased photosynthetic rates with a reduction in leaf carbohydrate content and an improvement in N assimilation. The increase in nitrate content was associated with up-regulation of genes of protein transcripts of photosynthesis and N assimilation that favoured plant performance under elevated CO_2_. Finally, decreasing the CO_2_ from 700 ppm to 400 ppm revealed that both stomatal closure and inhibited expression of light-harvesting proteins negatively affected photosynthetic performance and plant growth.

## Teaser

Barley plants with higher carbon sink capacity in the peduncle are able to maintain photosynthetic capacity by avoiding build-up of carbohydrates in the flag leaf.

## Introduction

Atmospheric carbon dioxide (CO_2_) has increased from around 280 ppm recorded at the beginning of the Industrial Revolution (1780) to approximately 400 ppm at present, and it is expected to increase to over 900 ppm by the end of the 21st century, depending on the climate-change emission scenario (IPCC, 2014). While it would be logical to assume enhanced photosynthetic assimilation in C_3_ plants due to the increase in CO_2_, several studies have shown that a build-up of leaf carbohydrate linked to higher CO_2_ availability might induce a reduction in carboxylation efficiency (Ainsworth *et al.*, 2007; Bloom *et al.*, 2010; Aranjuelo *et al.*, 2015). Prolonged exposure to elevated CO_2_ often induces stomatal closure with a consequent impact on CO_2_ diffusion into the chloroplast, which would partly explain the decline in photosynthetic carboxylation capacity (Xu *et al.*, 2016). Non-stomatal limitations can also lead to a down-regulation of photosynthesis due to a decrease in the amount and activity of Rubisco, and this is accompanied by the accumulation of carbohydrates. Several factors have been proposed to explain the phenomenon of CO_2_ acclimation, including insufficient plant sink strength (Moore *et al.*, 1999; Ainsworth and Rogers, 2007), nitrogen (N) dilution by accumulation of carbohydrates in leaves (Stitt and Krapp, 1999), and/or inhibition of nitrate assimilation (Bloom *et al.*, 2010). Enhanced leaf C content caused by greater photosynthetic rates in plants exposed to elevated CO_2_ could lead to repression of photosynthesis-related genes and to a down-regulation of photosynthetic capacity (Ainsworth *et al.*, 2004; Aranjuelo *et al.*, 2009, 2011, Vicente *et al.*, 2015, 2016). The build-up of leaf carbohydrate has been associated with the capacity to develop strong C sinks, such as developing organs (Lewis *et al.*, 2002; Aranjuelo *et al.*, 2013). Thus, the presence of higher C sink strengths could contribute to preventing photosynthetic down-regulation via a better redistribution and allocation of carbohydrates to the developing sinks under elevated CO_2_ conditions (Ainsworth *et al.*, 2004; Aranjuelo *et al.*, 2013). Plants with a greater capacity to remobilize the ‘extra’ photoassimilates to organs with a higher C demand could have a similar advantage.

Nitrogen assimilation has also been identified as a key process that influences photosynthetic performance under elevated CO_2_. Photosynthesis provides C skeletons for assimilating N into amino acids to form proteins and other nitrogenous compounds. The imbalance between C fixation and N assimilation has been suggested as the main factor responsible for photosynthetic down-regulation under elevated CO_2_ (Ainsworth and Long, 2005; Bloom *et al.*, 2010). In addition, limitations in N assimilation observed in plants grown under elevated CO_2_ have been associated with a reduction in energy availability, which would have effects on C and N metabolism (Rachmilevitch *et al.*, 2004; Bloom *et al.*, 2010; Aranjuelo *et al.*, 2013). Such limitations in energy availability would modify the C/N ratio by increasing the carbohydrate content and decreasing the N pool due to competition for reductant (Rachmilevitch *et al.*, 2004; Bloom *et al.*, 2010).

Assimilation and remobilization of C compounds is important during grain filling. To sustain grain filling in C_3_ cereals, photoassimilates are mainly provided from photosynthesis in the upper leaves, predominantly the flag leaf and penultimate leaves in barley and the flag leaf in wheat (Evans, 1983; Hosseini *et al.*, 2012; Liu *et al.*, 2015), from the remobilization of C stored in leaves and peduncles that was assimilated before anthesis (Gebbing and Schnyder, 1999), and from photosynthesis in the ear (Tambussi *et al.*, 2007; Zhou *et al.*, 2016). Sucrose, fructans, and starch are the most important carbohydrates that affect crop performance during the grain-filling period in barley. Sucrose is the major transported form of carbohydrate and it provides most of the energy and C necessary for the growth and development of non-photosynthetic organs. Together with starch, fructans have been described as the major C storage compounds in different cereal organs such as the grains, leaves, stems, and roots (Morcuende *et al.*, 2004). In addition to their role as reserve carbohydrates, fructans also provide C and energy to non-photosynthetic tissues when the C demand is high (Xue *et al.*, 2011; Van den Ende, 2013). In addition, carbohydrates can also act as signal molecules that regulate the expression of a wide variety of genes involved in different metabolic pathways and cellular functions (Osuna *et al.*, 2007; Van den Ende, 2013; Valluru, 2015). Fructan synthesis is activated when sucrose content exceeds a threshold concentration (Pollock and Cairns, 1991; Koroleva *et al.*, 1998). The increase in sucrose content increases fructosyltransferase gene expression, whereas high nitrate content inhibits its expression (Morcuende *et al.*, 2004). A close correlation between increases in carbohydrate content and the down-regulation of genes involved in photosynthesis and N metabolism has been recently reported (Vicente *et al.*, 2018).

In the search for more productive varieties, conventional plant-breeding programs have reduced the genetic diversity of crops by the use of ‘elite’ varieties that have lost alleles relevant to specific environmental conditions (Ellis *et al.*, 2000; Dawson *et al.*, 2015). The intensive plant-breeding programs conducted over recent decades have mainly been focused on the selection of genotypes with high harvest indices. However, such selection has not contributed to overcoming sink–source limitations (Maydup *et al.*, 2012; Schmid *et al.*, 2016). To recover some of the favourable alleles lost during plant-breeding programs, Matus *et al.* (2003) developed a recombinant chromosome substitution line (RCSL) population of 140 lines using the wild ancestor of barley, *Hordeum vulgare* subsp. *spontaneum*, as a source of donor alleles for *H. vulgare* subsp. *vulgare* cv ‘Harrington’, which is commonly used as a quality standard in North America. The recovered genes in the RCSL-89 line showed higher tolerance to abiotic stress by accumulating more carbohydrates under drought (Méndez *et al.*, 2011).

Adaptation of crops to future atmospheric conditions will require a better understanding of how plants respond to increasing C content. In this study, we took two approaches to investigate the importance of the C sink–source balance in the responsiveness of plants to different CO_2_ conditions. The first goal was to determine the relevance of the balance in response to elevated CO_2_. For this, barley genotypes with high (RCLS-89) and low (Harrington) capacities to store C/N compounds in the peduncles were exposed to elevated CO_2_. The second goal was to characterize and identify mechanisms developed by plants to regulate their C sink–source balance under changing CO_2_. For this, two sets of 64 plants were grown. The first set was initially exposed to ambient CO_2_ (400 ppm) followed by elevated CO_2_ (700 ppm), and the second set was initially exposed to elevated CO_2_ (700 ppm) followed by ambient CO_2_ (400 ppm). The comparisons made between the responses of elite cultivars and newly developed genotypes have implications for future breeding programs that seek adaptive alleles to maintain the C sink–source balance under changing CO_2_.

## Materials and methods

### Plant material and experimental design

Seeds of the two barley genotypes, *Hordeum vulgare* subsp. *vulgare* cv Harrington and RCLS-89 (Matus *et al.*, 2003), were kept at 4 °C for 10 d to synchronize germination. Once germinated, 64 plants were grown in 32 pots (6.7 l) filled with a mixture of perlite:vermiculite (1:2, v:v) in two controlled environment chambers (Phytotron Service, SGIker, UPV/EHU). The conditions inside the chambers were 550 µmol m^−2^ s^−1^ photosynthetic photon flux density (PPFD) and a 14/10 h light/dark regime at 25/17 °C and 50/60% relative humidity. The plants were watered twice a week with Hoagland’s solution (Arnon and Hoagland, 1939) and once a week with deionized water to avoid salt accumulation. The experimental set-up was designed as two experiments in parallel. For Experiment 1, 32 plants were grown at different CO_2_ concentrations, ambient (400 ppm) or elevated (700 ppm), for 11 weeks (Supplementary Fig. S1 at *JXB* online). For Experiment 2, a set of 16 plants that had been grown for 5 weeks at ambient CO_2_ (i.e. up to flag-leaf emergence) were then exposed for the following 6 weeks to elevated CO_2_ (400–700 treatment). The reverse conditions were applied to another set of 16 plants that had been grown at elevated CO_2_ for 5 weeks and which were then exposed to ambient CO_2_ for 6 weeks (700–400 treatment) (Supplementary Fig. S1).

### Biomass and gas-exchange measurements

At the end of both experiments (week 11), gas-exchange measurements were conducted, beginning 2 h after the start of the photoperiod. The measurements were made on the flag leaf of plants at the medium milk stage (Zadoks stage 75). The net photosynthetic rate (*A*_N_) was measured at 500 μmol m^−2^ s^−1^ PPFD together with the stomatal conductance (*g*_s_) and intercellular CO_2_ (*C*_i_) using a 6400-XT portable gas-exchange system (LI-COR Inc.). Curves of net CO_2_ assimilation rate versus intercellular CO_2_ concentration (*A*/*C*_i_) were obtained under saturated light (1000 μmol m^−2^ s^−1^ PPFD) with the following steps: 400, 300, 200, 100, 400, 500, 600, 800, 1000, 1200, 400 ppm CO_2_, with typically 2–3 min between each step. For the estimation of the maximum carboxylation velocity of Rubisco (*V*_cmax_) we used the equation developed by Sharkey *et al.* (2007).

After measuring photosynthesis, four plants from each treatment were harvested for biochemical and molecular analyses. The flag leaves and peduncles were immediately plunged into liquid N and stored at –80 °C until further analyses. For determination of biomass, the main stems and tillers of four other plants were dried in an oven at 80 °C for 72 h. The contribution of the ear biomass at the end of the experiment was calculated as (Ear Biomass/Total biomass) × 100 (%).

### Carbon and nitrogen contents

Flag leaves and peduncles dried at 80 °C for 72 h were ground and 1 mg of material per sample was loaded into small tin capsules analysed using a Flash 1112 Elemental Analyzer (Carbo Erba, Milan).

### Determination of metabolites

Frozen flag leaf and peduncle material was used for ethanol/water extraction for carbohydrate determination according to Morcuende *et al.* (2004). Sucrose, starch, and fructan contents were subsequently determined spectrophotometrically following the protocol described by Morcuende *et al.* (2004). In the flag leaf, total amino acids were determined using the ninhydrin method (Hare and Cress, 1997), ammonium quantification was carried out using the Berthelot method (Patton and Crouch, 1977) based on the phenol hypochlorite assay, and nitrate quantification was carried out according to nitration of salicylic acid as described by Cataldo *et al.* (1974).

### Soluble protein extraction and Rubisco quantification

Protein extraction from flag leaves was carried out according to Gibon *et al.*, (2004). Total soluble proteins were quantified spectrophotometrically using the Bradford dye-binding assay (Bio-Rad) with BSA as the standard for the calibration curve. For determination of relative Rubisco content (%), protein extracts were denatured at 95 °C for 5 min after adding one volume of loading buffer (Laemmli, 1970) and then 10 µg of denatured proteins were separated by SDS-PAGE in 10% acrylamide. Electrophoresis was carried out in a vertical electrophoresis cell (Mini-Protean III; Bio-Rad) at room temperature and at a constant current of 120 V for 2 h. The gels were stained with 1% Coomassie Blue solution for 1 h and subsequently destained by washing four times in water:methanol:acetic acid (4:4:2, v:v:v) for 20 min. The gels were scanned and the density of the Rubisco subunit band was determined using ImageJ software.

### N-assimilation enzyme activities

The maximum activity of nitrate reductase (NR) was determined as described by Baki *et al.* (2000). The reaction was incubated for 30 min at 30 °C after the addition of 50 µl of protein extract to 250 µl of reaction buffer (50 mM HEPES-KOH, pH 7.6, 10 mM FAD, 1 mM DTT, 5 mM KNO_3_, 0.2 mM NADH, and 20 mM Na_2_-EDTA). The reaction was stopped by adding 0.5 M zinc acetate and the samples were centrifuged at 4000 *g* for 30 min at 4 °C. For determination of nitrite, 1% sulfanilamide in 3 M HCl and 0.02% N-naphthyl-ethylenediamine hydrochloride (NEDA) were added and the resulting reaction was measured colorimetrically at 540 nm, with KNO_2_ used as the standard for the calibration curve. Glutamine synthetase (GS) activity was determined by incubating 50 µl of protein extract for 30 min at 30 °C with 100 µl reaction buffer (50 mM TRIS-HCl, pH 7.6, 20 mM MgSO_4_, 4 mM Na_2_-EDTA, 80 mM sodium glutamate, 6 mM hydroxylamine, and 8 mM ATP). The reaction was stopped by adding 150 µl of 0.122 M FeCl_3_, 0.5 M TCA, and 2 N HCl. Samples were then centrifuged at 2000 *g* for 5 min and the absorbance of γ-glutamylmonohydroxamate (γ-GHM) in the supernatant was measured at 540 nm, with γ-GHM used as the standard for the calibration curve. The activities of glutamate dehydrogenase (GDH) and glutamate synthase (GOGAT) were determined by changes in the NADH concentration at 340 nm in a reaction consisting of 20 µl protein extract and 280 µl of reaction buffer for NADH-dependent GDH (100 mM Tris–HCl, pH 8, 1 mM CaCl_2_, 13 mM 2-oxoglutarate, 50 mM (NH_4_)_2_SO_4_, and 0.25 mM NADH) or for NADH-dependent GOGAT (100 mM Tris–HCl, pH 8.6, 10 mM DTT, 1 mM 2-oxoglutarate, and 0.2 mM NADH).

### RNA extraction and synthesis of cDNA

RNA was isolated from pulverized frozen flag leaves using the phenol:chloroform method described by Morcuende *et al.* (1998). Then, 10 µg of RNA for each sample were treated with DNase Turbo (Ambion) according to the manufacturer’s instructions. RNA integrity was checked on a 1.5% (v/v) agarose gel and the absence of genomic DNA contamination was confirmed by PCR using a primer pair for the gene encoding glyceraldehyde-3-phosphate dehydrogenase (GenBank ID: EF409633) designed to amplify exon-intron-exon sequences with a product size of 120 bases for RNA and 360 bases for genomic DNA. cDNA was synthesized using SuperScript III reverse transcriptase (Invitrogen GmbH) according to the manufacturer′s instructions.

### Quantitative real-time PCR

Gene expression was measured as described by Vicente *et al.* (2015). Quantitative PCR was performed in an optical 384-well plate with a PRISM 7900 HT Sequence Detection System (Applied Biosystems) in a 10-µl reaction volume using the SYBR Green Maxter Mix reagent (Applied Biosystems), 1 µl of diluted cDNA (1:40), and 200 nM of each gene-specific primer. The PCR thermal profile was as follows: polymerase activation (50 °C for 2 min, 95 °C for 10 min) amplification and quantification cycles repeated for 40 cycles (95 °C for 15 s and 60 °C for 1 min), and a final step of 95 °C for 15 s and 60 °C for 15 s to obtain the dissociation curve. Three biological replicates were used for quantification analysis with two technical replicates for each biological sample. Transcript levels for genes associated with photosynthesis, carbohydrate metabolism, and N-assimilation in flag leaves were determined using the primers described in Méndez (2014) and Córdoba *et al.* (2016) (Supplementary Table S1). The data are presented as the log_2_-fold change after the quantification of the relative gene expression using the comparative *C*_T_ method (Schmittgen and Livak, 2008), and using *actin* as a reference for normalizing the gene expression results (Córdoba *et al.*, 2016).

### Statistical analysis

Data were analysed using the SPSS Statistics 22 software. Normality and homogeneity of variance were analysed using the Kolmogorov–Smirnov and Levene tests. Differences in the effects of CO_2_ on the two barley genotypes were determined by one-way ANOVA with a Duncan *post hoc* test. Differences between the two genotypes in the same CO_2_ conditions were analysed using Student’s *t*-test. For both analyses, differences were considered significant at *P*<0.05.

## Results

### Experiment 1: evaluation of the relevance of the plant C sink–source balance in responses to elevated CO_2_

**Fig. 1.**
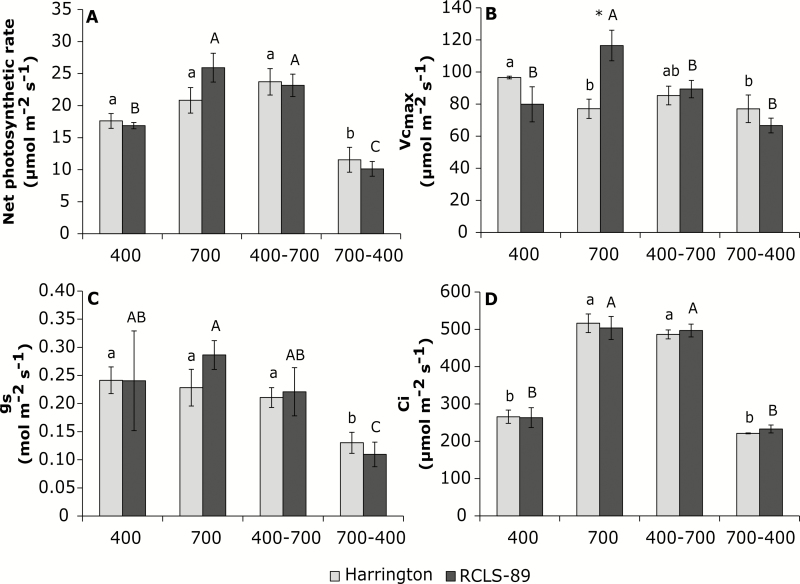
Effects of CO_2_ on flag-leaf gas-exchange and photosynthesis parameters for the barley genotypes Harrington and RCSL-89. (A) Net photosynthetic rate (*A*_N_), (B) maximum velocity of RuBP carboxylation by Rubisco (*V*_cmax_), (C) stomatal conductance (*g*_s_), and (D) intercellular CO_2_ concentration (*C*_i_). CO_2_ growth conditions were 400 ppm, 700 ppm, increase from 400 ppm to 700 ppm (400–700), and decrease from 700 ppm to 400 ppm (700-400). Significant differences (*P*<0.05) between each CO_2_ condition are indicated with different letters: lowercase letters indicate significant differences for Harrington and capital letters for RCSL-89. * Indicates significant genotype differences (*P*<0.05). Values are means (±SEM) of four biological replicates.

Exposure to elevated CO_2_ did not alter the total biomass but did increased the ear biomass in both barley genotypes, as shown by the higher ear biomass contribution relative to 400 ppm CO_2_ (Table 1). In the Harrington genotype, the photosynthetic rate (*A*_N_) at 700 ppm CO_2_ was not increased relative to 400 ppm (Fig. 1A) but the maximum carboxylation velocity of Rubisco (*V*_cmax_) was decreased (Fig. 1B), whilst the stomatal conductance (*g*_s_) was similar under both CO_2_ conditions (Fig. 1C). In RCSL-89 plants exposed to elevated CO_2_, *A*_N_ and *V*_cmax_ were increased relative to ambient CO_2_ (Fig. 1A, B). Comparing the Harrington and RCSL-89 plants, the results suggested a possible photosynthetic down-regulation in Harrington, because although there was no difference in the internal CO_2_ concentration (*C*_i_) of Harrington flag leaves relative to RCSL-89, the value of *V*_cmax_ was decreased at 700 ppm (Fig. 1B, D).

**Table 1.** Total biomass, ear biomass, and the ear biomass contribution (%) of the Harrington and RCSL-89 genotypes at the medium milk stage (Zadoks stage 75)

	CO_2_ conditions	Total biomass (g)	Ear biomass (g)	Ear biomass contribution (%)
Harrington	400	12.88 ± 1.38 ^a^	2.18 ± 0.40 ^b^*	0.17 ± 0.02 ^b^*
	700	13.15 ± 1.76 ^a^	3.77 ± 0.36 ^a^	0.33 ± 0.03 ^a^
	400–700	9.68 ± 0.81 ^ab^*	2.09 ± 0.27 ^b^	0.22 ± 0.02 ^b^
	700-400	8.52 ± 0.53 ^b^	1.98 ± 0.32 ^b^*	0.23 ± 0.04 ^b^
RCLS-89	400	11.40 ± 1.07 ^A^	1.05 ± 0.13 ^C^*	0.10 ± 0.01 ^C^*
	700	14.48 ± 1.39 ^A^	4.59 ± 0.19 ^A^	0.30 ± 0.02 ^A^
	400–700	12.72 ± 1.24 ^A^*	2.73 ± 0.21 ^B^	0.22 ± 0.02 ^B^
	700-400	13.62 ± 1.23 ^A^	3.81 ± 0.36 ^B^*	0.25 ± 0.03 ^AB^

CO_2_ conditions were 400 ppm, 700 ppm, increase from 400 ppm to 700 ppm (400–700), and decrease from 700 ppm to 400 ppm 700–400). Significant differences (*P*<0.05) between each CO_2_ condition are indicated with different letters: lowercase letters indicate differences for Harrington and capital letters for RCSL-89. * Indicates significant genotype differences. Values are means (±SEM) of 4 biological replicates.

**Fig. 2.**
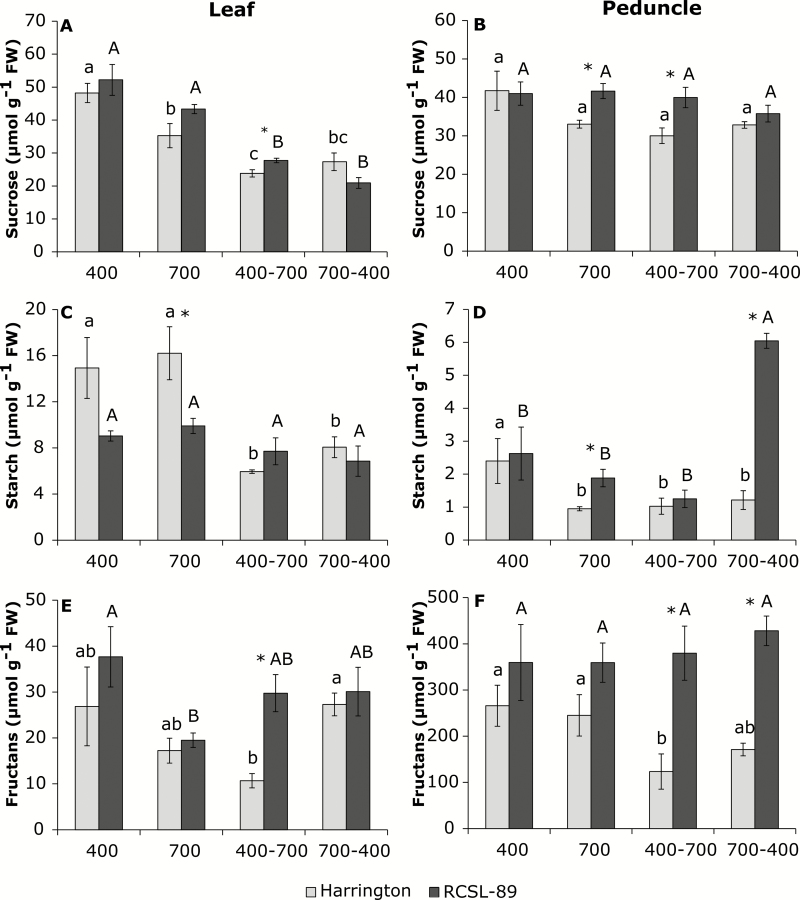
Effects of CO_2_ on contents of carbohydrates in the flag leaf and peduncle in the barley genotypes Harrington and RCSL-89. (A, B) Sucrose, (C, D) starch, and (E, F) fructans. CO_2_ growth conditions were 400 ppm, 700 ppm, increase from 400 ppm to 700 ppm (400–700), and decrease from 700 ppm to 400 ppm (700-400). Significant differences (*P*<0.05) between each CO_2_ condition are indicated with different letters: lowercase letters indicate significant differences for Harrington and capital letters for RCSL-89. * Indicates significant genotype differences (*P*<0.05). Values are means (±SEM) of four biological replicates.

In order to determine the level of photoassimilates and their mobilization to sink organs, the sucrose, starch, and fructan contents were determined in flag leaves and the peduncles of the two barley genotypes (Fig. 2). Flag leaves of Harrington grown under elevated CO_2_ had lower sucrose levels than under ambient CO_2_ (Fig. 2A), but did not show significant differences in starch and fructan contents (Fig. 2C, E). Elevated CO_2_ did not significantly alter the sucrose and starch contents in the flag leaves and peduncles of RCSL-89 plants (Fig. 2A–D), but it decreased the fructan content in the flag leaves (Fig. 2E); however, the fructan content of the peduncles was not significantly changed compared to plants grown at 400 ppm CO_2_ (Fig. 2F).

**Fig. 3.**
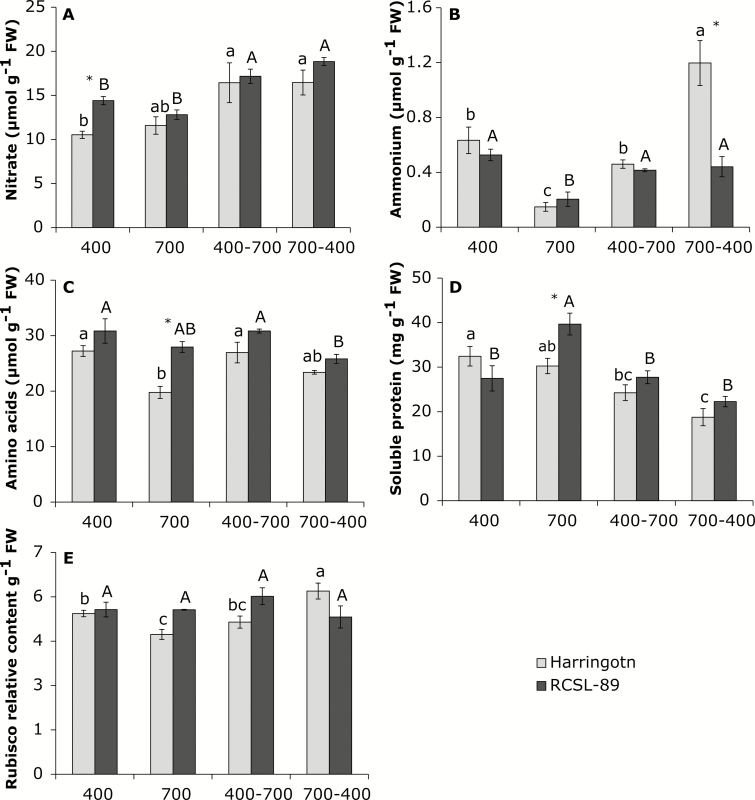
Effects of CO_2_ on N forms (nitrate and ammonium), amino acids, and soluble proteins in flag leaves of the barley genotypes Harrington and RCSL-89. (A) Nitrate, (B) ammonium, (C) amino acids, (D) soluble protein, and (E) relative Rubisco content. CO_2_ growth conditions were 400 ppm, 700 ppm, increase from 400 ppm to 700 ppm (400–700), and decrease from 700 ppm to 400 ppm (700-400). Significant differences (*P*<0.05) between each CO_2_ condition are indicated with different letters: lowercase letters indicate significant differences for Harrington and capital letters for RCSL-89. * Indicates significant genotype differences (*P*<0.05). Values are means (±SEM) of four biological replicates.

**Fig. 4.**
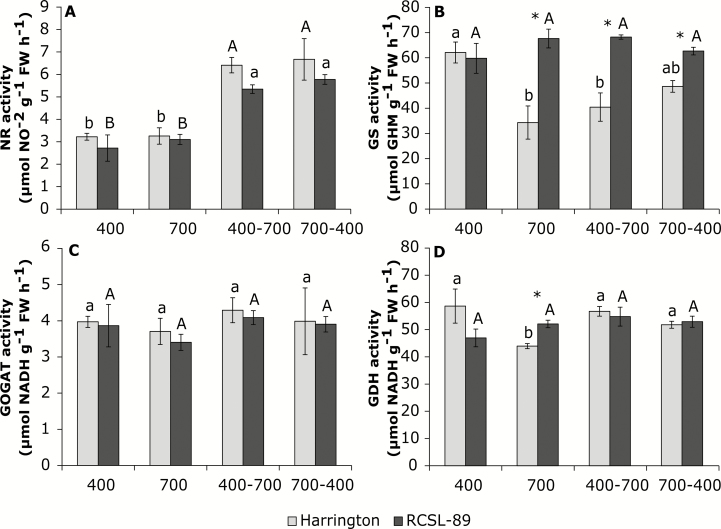
Effects of CO_2_ on flag leaf N enzyme activities in the barley genotypes Harrington and RCSL-89. (A) Nitrate reductase (NR), (B) glutamine synthetase (GS), (C) glutamate dehydrogenase (GOGAT), and (D) glutamate synthase (GDH). CO_2_ growth conditions were 400 ppm, 700 ppm, increase from 400 ppm to 700 ppm (400–700), and decrease from 700 ppm to 400 ppm (700-400). Significant differences (*P*<0.05) between each CO_2_ condition are indicated with different letters: lowercase letters indicate significant differences for Harrington and capital letters for RCSL-89. * Indicates significant genotype differences (*P*<0.05). Values are means (±SEM) of four biological replicates.

The C and N (%) contents in the flag leaves and peduncles of the two barley genotypes were not significantly affected by changes in the CO_2_ conditions (Table 2). Interestingly, the leaf C/N ratio was higher in RCSL-89 under elevated CO_2_, with a value that was similar to the flag leaves of Harrington (Table 2). The leaf content of N compounds such as nitrate, ammonium, total amino acids, and soluble proteins in the two genotypes showed different responses to exposure to elevated CO_2_ (Fig. 3). Although elevated CO_2_ did not reduce the amount of nitrate in flag leaves of either genotype, the ammonium content was decreased in both (Fig. 3A, B). Interestingly, the relative contents of amino acids and Rubisco in flag leaves of Harrington grown at 700 ppm CO_2_ were lower than at 400 ppm CO_2_, but no significant differences were observed in RCSL-89 (Fig. 3C, E). Prolonged exposure to elevated CO_2_ increased the foliar soluble protein content in RCSL-89 but not in Harrington (Fig. 3D). Exposure to elevated CO_2_ did not significantly affect the activities of NR and GOGAT in either of the two genotypes (Fig. 4A, C). However, elevated CO_2_ decreased the activities of GS and GDH in Harrington but not in RCSL-89, with the latter maintaining similar activities to plants grown at 400 ppm CO_2_ (Fig. 4B, D).

**Table 2.** N and C contents in the flag leaves and peduncles of the Harrington and RCLS-89 genotypes grown under different CO_2_ conditions

	CO_2_ conditions	Leaf C (%)	Leaf N (%)	Leaf C/N	Peduncle C (%)	Peduncle N (%)	Peduncle C/N
Harrington	400	45.19 ± 0.16 ^a^	3.47 ± 0.26 ^a^	13.18 ± 0.99 ^a^	42.36 ± 0.23 ^a^*	1.37 ± 0.99 ^a^	30.88 ± 0.50 ^b^
	700	44.57 ± 0.24 ^a^	3.00 ± 0.14 ^a^	14.91 ± 0.67 ^a^	42.39 ± 0.35 ^a^*	0.95 ± 0.67 ^ab^	45.32 ± 3.88 ^ab^
	400–700	43.47 ± 0.36 ^b^	3.16 ± 0.49 ^a^	14.53 ± 2.49 ^a^	44.60 ± 2.03 ^a^	0.90 ± 2.49 ^b^	51.21 ± 7.97 ^a^
	700-400	44.46 ± 0.36 ^a^	3.14 ± 0.25 ^a^	14.36 ± 1.33 ^a^	41.53 ± 0.10 ^a^*	1.17 ± 1.33 ^ab^	36.93 ± 7.08 ^ab^
RCLS-89	400	45.22 ± 0.71 ^A^	4.08 ± 0.47 ^A^	11.31 ± 1.01 ^B^	43.34 ± 0.05 ^A^*	1.34 ± 0.22 ^A^	34.12 ± 5.41 ^A^
	700	44.93 ± 0.29 ^A^	3.25 ± 0.12 ^AB^	13.84 ± 0.47 ^A^	43.52 ± 0.07 ^A^*	1.01 ± 0.11 ^A^	44.24 ± 5.18 ^A^
	400–700	43.53 ± 0.16 ^B^	4.08 ± 0.09 ^A^	10.67 ± 0.20 ^B^	42.95 ± 0.31 ^A^	1.36 ± 0.20 ^A^	33.10 ± 5.43 ^A^
	700-400	43.23 ± 0.31 ^B^	3.00 ± 0.20 ^B^	14.51 ± 0.94 ^AB^	42.79 ± 0.51 ^A^*	1.46 ± 0.30 ^A^	31.78 ± 6.06 ^A^

CO_2_ conditions were 400 ppm, 700 ppm, increase from 400 ppm to 700 ppm (400–700), and decrease from 700 ppm to 400 ppm 700–400). Significant differences (*P*<0.05) between each CO_2_ condition are indicated with different letters: lowercase letters indicate differences for Harrington and capital letters for RCSL-89. * Indicates significant genotype differences. Values are means (±SEM) of 4 biological replicates.

**Fig 5.**
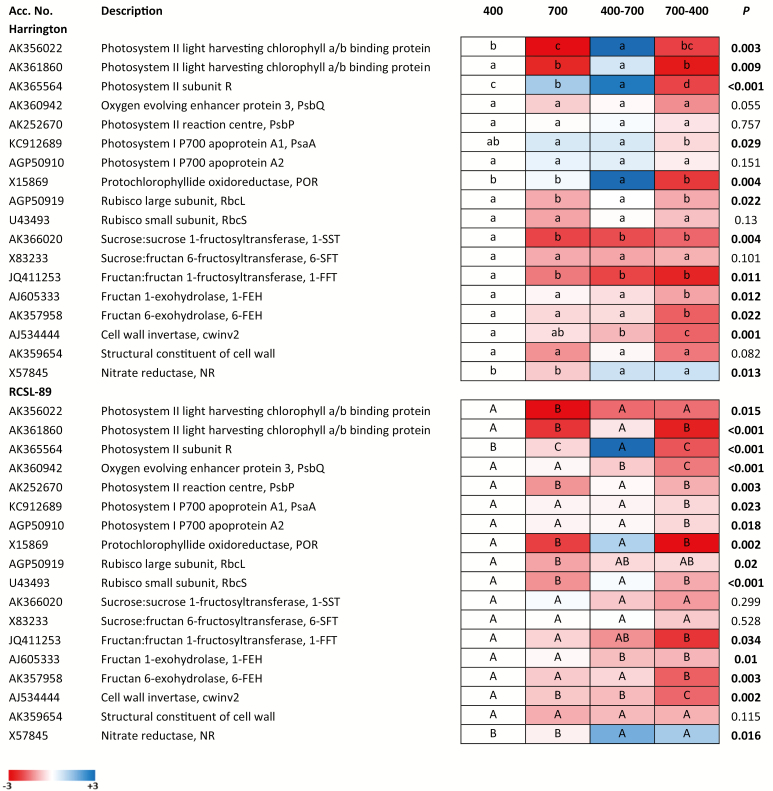
Heat map of the effects of CO_2_ on transcript abundance in leaves of Harrington and RCLS-89 barley genotypes. CO_2_ growth conditions were 400 ppm, 700 ppm, increase from 400 ppm to 700 ppm (400–700), and decrease from 700 ppm to 400 ppm (700–400). Significant differences (*P*<0.05) between each CO_2_ condition are indicated with different letters: lowercase letters indicate significant differences for Harrington and capital letters for RCSL-89. White indicates no change relative to the 400 ppm treatment, blue indicates up-regulation, and red indicates down-regulation, as shown in the log_2_ colour scale bar.

The relative expression of genes of both Harrington and RCSL-89 flag leaves are represented in Fig. 5. Exposure to elevated CO_2_ decreased the transcripts for photosynthetic proteins, including PSII light-harvesting chlorophyll *a*/*b* binding protein and the Rubisco large subunit in both genotypes, but it did not affect the transcripts for PSI-related genes. Exposure to elevated CO_2_ also significantly decreased the transcripts for the Rubisco small subunit in RCSL-89 but not in Harrington. In addition, elevated CO_2_ decreased the expression of the fructan-related genes 1-SST and 1-FFT in Harrington, but these were not changed in RCSL-89. Consistent with the lack of changes in NR activity previously observed (Fig. 4A), the gene expression for NR was not altered by elevated CO_2_, regardless of the genotype.

### Experiment 2: testing the plant mechanisms of response to changing CO_2_

#### Increasing CO_2_ from 400 ppm to 700 ppm

Increasing the CO_2_ concentration after 5 weeks of growth at 400 ppm to 700 ppm for a further 6 weeks (400–700) did not significantly decrease the total biomass of Harrington plants compared to those grown continuously at 700 ppm (Table 1). In addition, the ear biomass and the ear biomass contribution (%) in Harrington were similar to plants grown continuously at 400 ppm. On the other hand, while the total biomass of RCSL-89 was not affected when CO_2_ was increased (Table 1), the ear biomass contribution of these plants were increased, although not as much as in plants that were grown continuously in 700 ppm. The photosynthetic parameters in Harrington exposed to the increase in CO_2_ were similar to those grown continuously at 700 ppm, but *V*_cmax_ was lower in for the equivalent plants of RCSL-89 (Fig. 1A, B).

Increasing the CO_2_ reduced the sucrose and starch contents in Harrington flag leaves compared to plants that were grown continuously at 700 ppm CO_2_ (Fig. 2A, C, E). On the other hand, the flag leaves of RCSL-89 plants had lower sucrose content than those grown continuously at 700 ppm CO_2_, but showed similar starch and fructan contents. In the peduncles, the only change observed was that Harrington had a lower fructan content than plants grown continuously at 700 ppm CO_2_ (Fig. 2F). RCSL-89 maintained higher fructan contents than Harrington in the flag leaves and peduncles when the CO_2_ was increased (Fig. 2E, F).

Increasing the CO_2_ concentration reduced the C content of leaves of both genotypes relative to plants grown continuously at 700 ppm, but a similar effect did not occur in the peduncles (Table 2). The increase in CO_2_ did not result in any substantial changes in N content in Harrington flag leaves and peduncles (Table 2). The increase in CO_2_ resulted in increases in the ammonium and amino acid contents in Harrington flag leaves (Fig. 3B, C), whilst in RCSL-89 the nitrate and ammonium contents increased (Fig. 3A, B), but the amino acid content did not vary (Fig. 3C) and the protein content decreased relative to plants grown continuously at 700 ppm CO_2_ (Fig. 3D). Both genotypes showed higher NR activity when the CO_2_ was increased (Fig. 4A), but the activities of GS and GOGAT were unaffected relative to continuous growth at 700 ppm (Fig. 4B, C). GDH had different patterns of activity, increasing in flag leaves of Harrington but remaining unaltered in those of RCSL-89 (Fig. 4D).

Flag leaves showed differences in transcriptional responses to the increase in CO_2_ as several photosynthetic genes were induced in both genotypes Fig. 5). Specifically, transcripts of PSII light-harvesting proteins, the Rubisco large subunit, and protochlorophyllide oxidoreductase were induced in Harrington plants compared to those grown continuously at 700 ppm CO_2_. The gene encoding NR was also up-regulated in both genotypes, whilst the gene encoding fructan 1-exohydrolase was repressed in RCSL-89.

#### Decreasing CO_2_ from 700 ppm to 400 ppm

Decreasing the CO_2_ concentration after 5 weeks of growth at 700 ppm to 400 ppm for a further 6 weeks (700–400) resulted in Harrington having lower biomass compared to plants grown continuously at 400 ppm. However, both the ear biomass and the ear biomass contribution (%) were similar for 700-400 and 400 ppm plants (Table 1). Decreasing the CO_2_ did not significantly affect the total biomass of RCSL-89, but it did result in higher ear biomass contribution relative to plants grown continuously at 400 ppm (Table 1). In both genotypes, the reduction in CO_2_ decreased *A*_N_ and this was coupled with strong stomatal closure compared to plants grown at 400 ppm CO_2_ (Fig. 1A, C). *V*_cmax_ was significantly decreased in Harrington but not in RCSL-89 (Fig. 1B).

Decreasing the CO_2_ reduced the sucrose content in the flag leaves of both genotypes compared to those from plants grown continuously at 400 ppm (Fig. 2A), but it did not significantly change the sucrose content in the peduncles (Fig. 2B). The starch content in Harrington flag leaves and peduncles was reduced, whilst in RCSL-89 the starch content in the flag leaves did not vary but it increased in the peduncles in comparison to plants grown at 400 ppm CO_2_ (Fig. 2C, D). Neither the flag leaves nor peduncles of either Harrington or RCSL-89 plants showed significant differences in fructan content relative to plants grown continuously at 400 ppm (Fig. 2E, F). Corresponding with the decline in *A*_N_, decreasing the CO_2_ reduced the C content in flag leaves of both genotypes compared with plants grown continuously at 400 ppm (Table 2).

Decreasing the CO_2_ increased the nitrate and ammonium contents but reduced the protein content in Harrington (Fig. 3A, B, D). In RCSL-89, the content of nitrate increased, amino acids decreased, and proteins were unaltered relative to plants grown continuously at 400 ppm CO_2_ (Fig. 3A, C, D). The decrease in CO_2_ led to an increase in NR activity in both genotypes (Fig. 4A) but did not significantly affect the activities of the other enzyme studied (Fig. 4B–D).

Decreasing the CO_2_ repressed several genes that encode photosynthetic proteins (light-harvesting and the Calvin–Benson cycle) as well as genes involved in fructan metabolism (1-FFT, 1-FEH, and 6-FEH) and cell wall synthesis in the flag leaves of both Harrington and RCSL-89 compared with plants that were grown continuously at 400 ppm CO_2_ (Fig. 5). Decreasing the CO_2_ induced the gene encoding the NR enzyme.

## Discussion

The sink–source balance has been postulated as being key to conditioning the responsiveness of photosynthetic capacity to increasing CO_2_ (Ainsworth and Long, 2005; Aranjuelo *et al.*, 2011, 2013). In the present study two approaches were used to test the relevance of the capacity of the peduncle to accumulate carbohydrates, and the ‘plasticity’ of leaf C/N metabolism following modifications in the CO_2_ conditions.

### Experiment 1: a higher peduncle C-storage capacity contributes to overcoming photosynthetic down-regulation under elevated CO_2_

Carbon sink–source imbalance has been suggested as being responsible for the photosynthetic down-regulation frequently observed when plants are exposed to elevated CO_2_ (Aranjuelo *et al.*, 2011, 2013; White *et al.*, 2016). Indeed, an insufficient demand for carbohydrates from developing C-sinks has been observed to induce leaf C imbalances (White *et al.*, 2016). The peduncle has a special importance in the C-storage capacity for maintaining leaf C balance during the vegetative stage in cereals (Tambussi *et al.*, 2007). Later, during the grain-filling period, the C stored in the peduncle is remobilized towards the grain. In our study it was notable that in both barley genotypes, higher fructan contents were found in peduncles than in flag leaves (Fig. 2), showing the importance of these organs for the subsequent grain filling-stage.

Inadequate C-sink strength can lead to a decrease in photosynthetic activity so that C-source activity and sink capacity are balanced (White *et al.*, 2016). Exposure to elevated CO_2_ decreased *V*_cmax_ and the relative Rubisco content in Harrington (which has a low capacity to store C/N compounds in the peduncles), while an increase in *V*_cmax_ was found in RCSL-89 (high capacity to store C/N compounds in peduncles) (Fig. 1). The decline observed in Harrington was consistent with the photosynthetic down-regulation response widely observed under elevated CO_2_ (Pérez *et al.*, 2005; Aranjuelo et al., 2011, 2013; Vicente *et al.*, 2015). In addition, the depletion in Rubisco content in these plants, together with the decreases in amino acids and soluble proteins (Fig. 3), reduced the levels of leaf organic-N compounds, as has been observed in previous studies (Bloom *et al.*, 2002; Pérez *et al.*, 2005; Aranjuelo *et al.*, 2011; Vicente *et al.*, 2015). In contrast to Harrington, elevated CO_2_ led to an increase in the soluble protein content in RCSL-89 (Fig. 3). This suggested an improvement in leaf organic-N compounds that could have helped to maximize photosynthetic capacity, which is consistent with the higher *V*_cmax_ observed under elevated CO_2_ in RCSL-89 (Fig. 1). In addition, the drastic increase in ear biomass under elevated CO_2_ in RCSL-89 (Table 1) indicated that the strong sink capacity of this organ was especially important in the photosynthetic performance of this genotype under elevated CO_2_. The distribution of photoassimilates from flag leaves to the peduncles may have contributed to the avoidance of carbohydrate build-up under elevated CO_2_. Our results suggested that the improved leaf C balance in RCSL-89 may have helped to maintain N status, and consequently plants avoided photosynthetic down-regulation under elevated CO_2_. Similar to effects observed previously in wheat (Vicente *et al.*, 2015), in Harrington the down-regulation of genes encoding the Rubisco large subunit, together with decreased transcripts for proteins involved in light-harvesting (Fig. 5) and the lower Rubisco content under elevated CO_2_ (Fig. 3), may have contributed to the photosynthetic acclimation that we found in this genotype. The fact that at 700 ppm CO_2_ Harrington had a higher starch content than RCSL-89 (Fig. 2) may indicate that the flag leaves of Harrington were subjected to C sink–source imbalance. It should be noted that starch has been proposed as a way to store excess C in plants, while leaf sucrose content is suggested to represent the main form of C translocated towards developing sinks (Stitt *et al.*, 2010). This highlights the fact that impaired N assimilation, and consequently reduced Rubisco protein availability, could be linked to leaf C sink–source imbalances (Ainsworth *et al.*, 2004; Aranjuelo *et al.*, 2011, 2013; White *et al.*, 2016).

In our study, NR activity was not significantly affected by elevated CO_2_ in the flag leaves of either Harrington or RCSL-89 plants (Fig. 4). This suggested that CO_2_ enrichment did not restrict leaf nitrate reduction, which is in contrast with decreases reported in other species (Bloom *et al.*, 2002, 2010; Vicente *et al.*, 2015). The higher sucrose content in RCSL-89 could have contributed to the maintenance of NR expression and activity (Morcuende *et al.*, 1998) and to sustaining the activity of GS (Robredo *et al.*, 2011), with a consequent impact on amino acid and protein availabilities under elevated CO_2_ (Fig. 3). However, GS has been described as a target enzyme involved in N and C metabolism (Vega-Mas *et al.*, 2015). The decline in GS and GDH activities decreased the nitrate-assimilation pathway in Harrington flag leaves, which in turn altered the contents of amino acids and other organic-N compounds under elevated CO_2_. In contrast, the maintenance of the activities of these enzymes observed in RCSL-89 flag leaves would guarantee assimilation of inorganic nitrogen into amino acids. Indeed, total soluble protein levels increased in RCSL-89 flag leaves under exposure to elevated CO_2_ (Fig. 3). These findings suggest that a limitation in N assimilation could be involved in the decline in organic-N compounds and the down-regulation of photosynthetic capacity found in Harrington plants under elevated CO_2_. The improved photosynthetic acclimation responses to elevated CO_2_ in the RCSL-89 genotype were associated with enhanced flag-leaf N assimilation and a consequent increase in organic N compounds (Fig. 3). Moreover, the higher sink capacity of the peduncle and the ears would have facilitated the correct leaf C/N balance and overcome the photosynthetic down-regulation due to elevated CO_2_, confirming the importance of C-sink strength for increased crop yields under elevated CO_2_.

### Experiment 2: a balance in C and N metabolism modulates adaptability to changing CO_2_

As observed in Experiment 1, photosynthesis in plants grown under elevated CO_2_ is limited by the ability to adjust photosynthetic activity according to leaf C demand (Ziska *et al.*, 2004). To evaluate the adaptation capacity of the two barley genotypes to changing environmental CO_2_, plants were grown under ambient CO_2_ (400 ppm) or elevated CO_2_ (700 ppm) and then exchanged between treatments.

Increasing the CO_2_ concentration caused similar responses in *A*_N_, *g*_s_, *C*_i,_ and relative Rubisco content in both genotypes relative to plants grown continuously at 700 ppm (Figs 1, 3). Harrington maintained its photosynthetic capacity compared to plants grown continuously at 700 ppm CO_2_. However, the increase in *V*_cmax_ observed in RCSL-89 grown continuously at 700 ppm was not detected when it was initially grown at 400 ppm (Fig. 1). The ability to overcome photosynthetic acclimation may be linked to the up-regulation of genes encoding proteins involved in light-harvesting and the maintenance of Rubisco gene expression and protein content (Vicente *et al.*, 2015). Hence, our findings suggested that Harrington plants did not suffer photosynthetic down-regulation or, at least, that they showed a better photosynthetic capacity than RCSL-89 under increasing CO_2_.

In agreement with previous findings by Stitt *et al.* (2010), the higher starch content observed in Harrington compared to RCSL-89 grown under elevated CO_2_ (Fig. 2) could be considered a symptom of C overflow due to the rate of photosynthesis exceeding the rate of leaf C demand. This imbalance may have been associated with the down-regulation of amino acid storage, in agreement with previous studies (Yamakawa and Hakata, 2010; Midorikawa *et al.*, 2014). Interestingly, the starch content did not differ in the peduncles of either genotype after increasing the CO_2_ concentration, but RCSL-89 showed higher storage capacity for fructans in the flag leaves and peduncles than Harrington.

Increasing the CO_2_ increased the activity of NR in flag leaves of both Harrington and RCSL-89 relative to plants grown continuously at 700 ppm (Fig. 4). This indicated that the reduction of leaf nitrate was unaffected by CO_2_ enrichment, which is in contrast to the reduction in the N pool reported in other species grown under elevated CO_2_ (Bloom *et al.*, 2002, 2010; Vicente *et al.*, 2015). More notably than in the first experiment, CO_2_ enrichment induced the expression of NR genes and increased the nitrate content, as has been observed previously (Stitt and Krapp, 1999; Vicente *et al.*, 2016), while increasing the amino acid content and reducing the sucrose and starch contents relative to plants grown at 700 ppm CO_2_. The competition for reductants in the chloroplast stroma has been described as a factor that conditions C and N assimilation (Rachmilevitch *et al.*, 2004; Vicente *et al.*, 2016). For this reason, the leaf light-harvesting complexes and proteins involved in electron transport may be particularly important in maintaining the energy necessary for balancing both N and C metabolism. In agreement with Vicente *et al.* (2017), we observed that exposure to elevated CO_2_ induced the expression of PSII light-harvesting complexes (Fig. 5). More than 50% of the N that is assimilated by roots is allocated to flag leaves and comprises Rubisco, light-harvesting complexes, and other proteins involved in electron transport (Kitaoka and Koike, 2004). Our results suggested that increasing CO_2_ from 400 ppm to 700 ppm caused concomitant increases in *A*_N_ and nitrate content (Fig. 1, Table 2) and reductions in carbohydrate content (Table 2) by increasing energy availability for co-ordinating C and N assimilation under elevated CO_2_. These findings suggest that this stimulation of N assimilation could be involved in the increase in the amino acid content and the capacity to overcome the initial photosynthetic down-regulation found in Harrington under elevated CO_2_.

Decreasing the CO_2_ concentration from 700 ppm to 400 ppm after flag-leaf emergence caused significant stomatal closure and reduced photosynthetic rates (Fig. 1), which were associated with lower biomass in Harington plants (Table 1). Stomatal limitations are one of the mechanisms responsible for photosynthetic down-regulation under elevated CO_2_ (Xu *et al.*, 2016). Bloom *et al.* (2002, 2010) reported that a reduction in *A*_N_ increases nitrate assimilation because NR has access to a larger amount of NADH for reducing nitrate to nitrite. Our results suggested that plants exposed to decreasing CO_2_ suffered energy limitations due to a lower expression of light-harvesting complexes and reaction centres when compared to plants grown continuously at 400 ppm CO_2_ (Fig. 5). This photosynthetic limitation was reflected by a decrease in the leaf carbohydrate contents (Fig. 2). However, the peduncles of RCSL-89 plants showed a greater accumulation of starch, which is associated with long-term carbohydrate storage. In accordance with the photosynthetic limitations, genes related to photosynthesis, such as light-harvesting, Rubisco, and chlorophyll synthesis, were down-regulated, or at least showed similar expression to plants grown at 700 ppm (Fig. 5). Comparing Harrington and RCSL-89, the higher fructan content in the peduncles of RCSL-89 could have been linked to the repression of fructosyltransferases (particularly sucrose:sucrose 1-fructosyltransferase), which are involved in fructan synthesis. The lower sucrose and starch-storage capacity in flag leaves, together with the accumulation of fructans in the peduncles, revealed that the lower photosynthetic capacity acted to modify the C/N balance. In this regard, the lower biomass, especially in terms of ear weight, together with the lower levels of amino acids and proteins as well as the down-regulation of photosynthesis-related genes suggested that the plants attempted to adapt to the new environment.

## Conclusions and future perspectives

Our study has highlighted the importance of the C/N balance as influenced by photosynthesis and N assimilation in two barley genotypes exposed to elevated CO_2_, and the relevance of the peduncle sink capacity to this balance. Our study showed that in genotype Harrington, which has a low capacity to store C/N compounds in the peduncles, CO_2_ enrichment decreased photosynthetic capacity whereas genotype RCSL-89, which has a higher capacity, could overcome photosynthetic down-regulation under elevated CO_2_. The larger C-sink capacity of RCSL-89 enabled the avoidance of a build-up of leaf carbohydrates and enabled the maintenance of Rubisco protein in the flag leaves. On the other hand, the leaf C sink–source imbalance of Harrington plants grown under elevated CO_2_ was linked to a depletion of Rubisco content and a consequent decrease in *V*_cmax_. The increased expression of transcripts associated with light-harvesting complexes and changes to CO_2_ diffusion were shown to be significant in influencing plant growth and C and N metabolism when the CO_2_ conditions were modified. Increasing CO_2_ from 400 ppm to 700 ppm reduced leaf carbohydrate contents and improved N assimilation. On the other hand, decreasing the CO_2_ from 700 ppm to 400 ppm led to both stomatal closure and repression of transcripts of light-harvesting proteins, showing them to be the main factors involved in the inhibition of photosynthetic machinery and plant growth.

Within the context of current environmental conditions and those projected for the coming decades, it is crucial to increase crop yields through the development of cultivars that are resilient to environmental changes. While the work now underway in plant breeding programs is of great importance, there is a risk that alleles crucial to adaptations to this unpredictable future might be lost when selecting modern elite crop cultivars. Moreover, as evidenced here and in earlier studies, the introgression of genes from wild genotypes into modern varieties could overcome some sink–source limitations. Together, our results show that the RCSL-89 barley line has a greater responsiveness to elevated CO_2_ than the elite Harrington cultivar, highlighting the potential contribution that adaptive alleles from wild genotypes have to breeding programs.

## Supplementary data

Supplementary data are available at *JXB* online.

Table S1. List of primers used for real-time qPCR.

Fig. S1. Schematic diagram of the experimental regimes showing treatments and phenological stages.

## Supplementary Material

Supplementary Material
