# Supplementary material for: C and N metabolism in barley leaves and peduncles modulates responsiveness to changing CO2
Source: J Exp Bot. 2018 Nov 24;70(2):599–611. doi: 10.1093/jxb/ery380 (PMC6322569; doi:10.1093/jxb/ery380)
Supplement: Supplementary Material [file ery380_suppl_supplementary_material.pdf]

Table S1 Primer pairs for barley sequences associated with photosynthesis, carbohydrate metabolism and nitrogen assimilation.

**Acc. No.**	**Description**		**Sequence**	**Product (bp)**	**Reference**
AY145451	Actin (reference gene)	Fw	GGCACACTGGTGTCATGG	134	(Córdoba *et al.*, 2016)
		Rv	CTCCATGTCATCCCAGTT		
AK356022	Photosystem II light harvesting chlorophyll a/b binding protein	Fw	CATCCCCTCACGGCTTTCTT	67	(Córdoba *et al.*, 2016)
		Rv	CGCCGCCATTGTAGAGCTAA		
AK361860	Photosystem II light harvesting chlorophyll a/b binding protein	Fw	CGCCACCAACTTTGTTCCTG	147	(Córdoba *et al.*, 2016)
		Rv	ATCGAAGGCGGGCAAATCTT		
AK365564	Photosystem II subunit R	Fw	GCGGATTATAACCGTCAGGACA	140	(Córdoba *et al.*, 2016)
		Rv	TGTGAGAGAGCTTAGCACTGAA		
AK360942	Oxygen evolving enhancer protein 3, PsbQ	Fw	AAAGGGGACTACGCAGAAGC	73	(Córdoba *et al.*, 2016)
		Rv	AGCTCTTGATCCGGCAAACA		
AK252670	Photosystem II reaction center, PsbP	Fw	GACCTAGGCCCTCCTGAGAA	141	(Córdoba *et al.*, 2016)
		Rv	ATAGAGCTTGCCATCGTCCG		
KC912689	Photosystem I P700 apoprotein A1, PsaA	Fw	CGCAAGGAAAGCGAAAACCT	62	(Córdoba *et al.*, 2016)
		Rv	ATTTGCTCGGAGTTCCCGTT		
AGP50910	Photosystem I P700 apoprotein A2	Fw	CATTGAAAGCGGGGCCATTC	68	(Córdoba *et al.*, 2016)
		Rv	TGCTCATGGCAAGACGACAT		
X15869	Protochlorophyllide oxidoreductase, POR	Fw	CGTGTACTGGAGCTGGAACA	100	(Córdoba *et al.*, 2016)
		Rv	GGATTTGCGGTGGATCATGC		
AGP50919	Rubisco large subunit, RbcL	Fw	ACGTGCTCTACGTTTGGAGG	65	(Córdoba *et al.*, 2016)
		Rv	GCGGGCCTTGGAAAGTTTTT		
U43493	Rubisco small subunit, RbcS	Fw	ACCAACATGCTCGAGAAAGCA	141	(Córdoba *et al.*, 2016)
		Rv	GTGTGGGCGTGCAAAGATGT		
AK366020	Sucrose:sucrose 1-fructosyltransferase, 1-SST	Fw	GGCCAGGAAACAATCTACCCA	87	(Córdoba *et al.*, 2016)
		Rv	GGGATGAGAATGACGCGAGA		
X83233	Sucrose:fructan 6-fructosyltransferase, 6-SFT	Fw	CGTATCAGGAGGCAAGAGTC	98	(Méndez, 2014)
		Rv	GTTGTGTGCCGAGTCCAT		
JQ411253	Fructan:fructan 1-fructosyltransferase, 1-FFT	Fw	GACCGGCGAGACTATTACGC	110	(Méndez, 2014)
		Rv	CTGCCATAGTCGTAGCGCA		
AJ605333	Fructan 1-exohydrolase, 1-FEH	Fw	GGATTACGGCATTTCTACGC	69	(Méndez, 2014)
		Rv	CCCCATACAATCCTCCTGCC		
AK357958	Fructan 6-exohydrolase, 6-FEH	Fw	GTCAGAGGAGGAACTACGCAC	70	(Córdoba *et al.*, 2016)
		Rv	GGCGGTCGAGTCTGTCATAA		
AJ534444	Cell wall invertase, cwinv2	Fw	AGACGTTGAGGAGCAAACGA	140	(Méndez, 2014)
		Rv	GGTTTCTGCCTCTTCCAGGG		
AK359654	Structural constituent of cell wall	Fw	CATAGATCGAGCGGTGGCTA	75	(Córdoba *et al.*, 2016)
		Rv	AATCCGGGCCATCATGCTC		
X57845	Nitrate reductase, NR	Fw	ACCAACTGCGTCATCACCAC	135	(Méndez, 2014)
		Rv	GGATGGATGGATGGAGGAGGA		

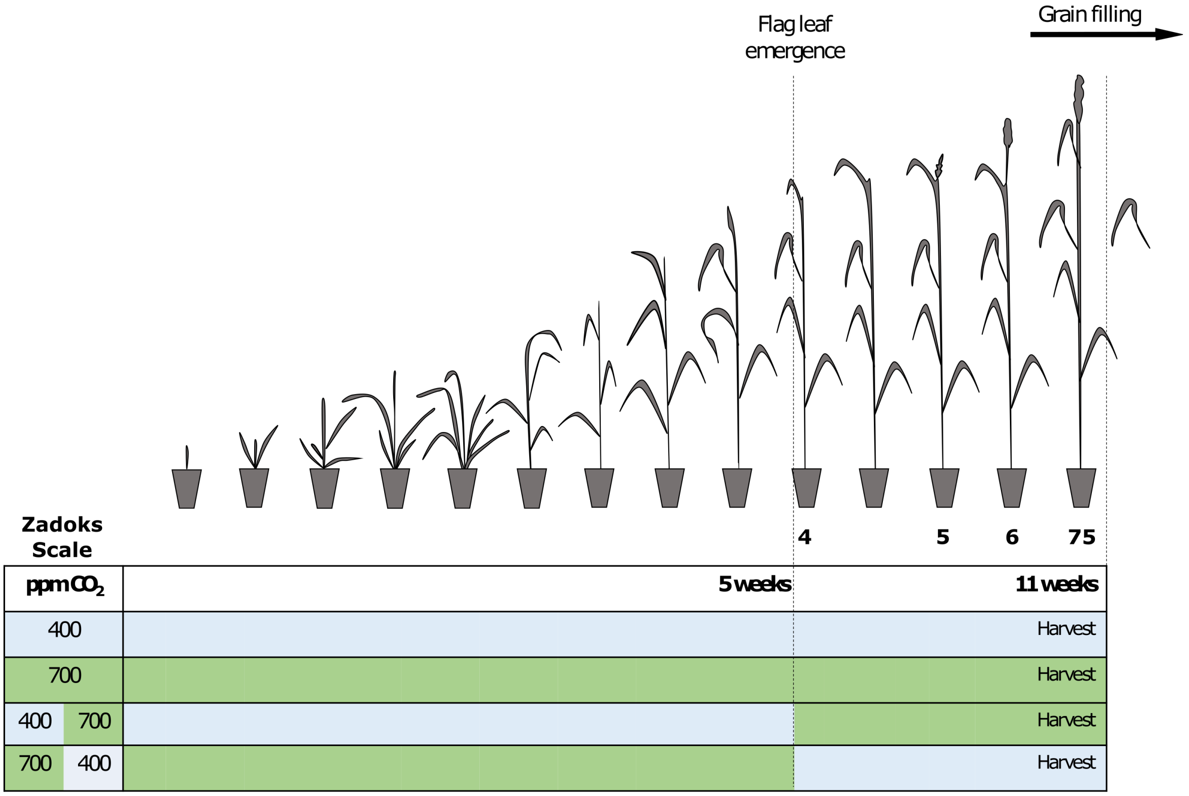

550 µmol m^{-2} s^{-1} photosynthetic photon flux density (PPFD)
14 h-light/10 h-darkness photoperiod; 25/17 °C and 50/60% relative humidity

Figure S1. Phenological stage and treatment chronogram.
